# Abdominal compression as motion management for stereotactic radiotherapy of ventricular tachycardia

**DOI:** 10.1016/j.phro.2023.100499

**Published:** 2023-10-07

**Authors:** Annika Mannerberg, Martin P. Nilsson, Anneli Edvardsson, Kristin Karlsson, Sofie Ceberg

**Affiliations:** a Medical Radiation Physics, Department of Clinical Sciences Lund, Lund University, Lund, Sweden; b Department of Hematology, Oncology and Radiation Physics, Skåne University Hospital, Lund, Sweden; c Division of Oncology and Pathology, Department of Clinical Sciences, Lund University, Lund, Sweden; d Radiation Physics, Department of Hematology, Oncology and Radiation Physics, Skåne University Hospital, Lund, Sweden; e Karolinska University Hospital, Section of Radiotherapy Physics and Engineering, Department of Medical Radiation Physics and Nuclear Medicine, Stockholm, Sweden; f Karolinska Institutet, Department of Oncology-Pathology, Stockholm, Sweden

**Keywords:** Stereotactic body radiotherapy for ventricular tachycardia, Motion management, Abdominal compression, Heart motion reduction

## Abstract

**Background and purpose:**

Stereotactic body radiotherapy (SBRT) has emerged as a promising treatment for patients with ventricular tachycardia (VT) who do not respond to standard treatments. However, the management of respiratory motion during treatment remains a challenge. This study aimed to investigate the effect of abdominal compression (AC) on respiratory induced motion in the heart.

**Materials and methods:**

A patient cohort of 18 lung cancer patients was utilized, where two four-dimensional computed tomography (4DCT) scans were performed for each patient, one with and one without AC. The patient setup consisted of an AC plate together with a stereotactic body frame. The left coronary artery, the left anterior descending artery, the lateral wall of the left ventricle, the heart apex, the carina, and the right and left diaphragm were delineated in max expiration and max inspiration phases in both 4DCT scans. The center of mass shift from expiration to inspiration phase was determined to assess the AC’s impact on respiratory motion.

**Results:**

A significant reduction in motion in the superior-inferior direction was found for all heart structures when AC was used. The median respiratory motion of the heart structures decreased by approximately 1–3 mm with AC in the superior-inferior direction, and approximately 60% of the patients had a motion reduction ≥3 mm in the left ventricle wall.

**Conclusion:**

These findings suggest that AC has the potential to improve the motion management of SBRT for VT patients, by reducing the respiratory induced motion in the heart.

## 1 Introduction

Ventricular tachycardia (VT) is a life-threatening condition characterized by a heart rate above 120 beats per minute triggered from a focus located in the left or right ventricle. Most patients suffering from VT have an underlying heart disease [1]. Standard treatments for VT include catheter ablation and anti-arrhythmic medications [1]. In recent years, stereotactic body radiotherapy (SBRT) has emerged as a promising treatment option for VT patients who do not achieve disease control with the standard treatments. By delivering 25 Gy in one fraction to the VT substrate (most commonly located in the left ventricle wall), Cuclich et al. [2] reported that VT episodes were reduced with 99.9% over the 46 months after treatment and Neuwirth et al. [3] found a reduction of 87.5% in VT burden at the median follow-up time of 28 months.

Generally, different imaging modalities are used for target delineation. Electrophysiological imaging of the heart is done either with electro-anatomical mapping or non-invasive electrocardiographic imaging [4], [5]. These images are combined with magnetic resonance (MR), computed tomography (CT) and/or SPECT or PET imaging. To assess respiratory and cardiac motion, a four-dimensional CT (4DCT) correlated to respiratory and cardiac phases respectively can be acquired [4]. All acquired images are used to localize and characterize the VT substrate, which constitutes the clinical target volume (CTV). The final planning target volume (PTV) encompasses all uncertainties such as motion and setup errors [6]. Hence, the PTV includes healthy tissue and may, depending on location, overlap with adjacent organs at risk (OAR). To avoid overlap with OAR it would be desirable to keep the PTV margin as small as possible. This would also be beneficial for possible retreatment. Haskova et al. [7] reported on reirradiation of three VT patients. The patients had no side effects, which the authors meant could be related to the fact the treated volumes were kept as small as possible, by tracking respiratory motion with the implantable cardioverter defibrillator (ICD) lead and including only cardiac motion in the PTV.

To minimize the irradiated volume, it is essential to decrease the motion of the heart. Hence, various motion management techniques should be evaluated for their potential to reduce motion. A common technique for respiratory motion reduction is abdominal compression (AC) [8]. It has previously been shown that AC has the potential to reduce tumour motion in thoracic and abdominal tumour sites [9], [10], [11], [12], [13], [14], [15], [16]. However, AC is not guaranteed to reduce respiratory motion. In lung cancer patients, Bouilhol et al. [16] demonstrated a significant motion reduction only for lower lobe lung tumours and Javadi et al [17] showed that AC reduced lung tumour motion in 5 out of 44 lesions. Van Gelder et al. [18] observed no statistically significant difference in motion of the kidneys and liver, comparing with and without AC. These studies also demonstrated an increase in motion due to AC for some patients [16], [17], [18]. The number of studies evaluating the impact of AC on respiratory induced heart motion is limited. Rasheed et al. [19] showed no statistically significant decrease in the motion of the whole heart using AC for lung cancer patients. However, the potential benefits or disadvantages of AC for reducing respiratory induced motion of the heart have not yet been thoroughly investigated. The aim of this study was therefore to investigate the effect of AC on motion in different structures in the heart and surrounding tissues, as a first step in optimising the motion management for SBRT of VT patients.

## 2 Materials and methods

### 2.1 Patients

Due to the limited number of VT SBRT patients in this early stage, a surrogate patient cohort was utilised instead. Thirty lung cancer patients were retrospectively included in this study. The study was approved by the Swedish ethical review authority (No Dnr 2022-02382-02) and all patients signed informed consent. The patients had all undergone two 4DCT scans, one with AC and one without AC. After review of the data 12 of the patients were excluded due to either large motion artefacts in the 4DCT images or the heart not being fully included in the scanned volume. Characteristics of the included patients are presented in Table S-1 in Supplementary materials. For the 18 eligible patients the median age was 75.5 years (range: 51–81 years) and the patients consisted of 11 women and 7 men. Fourteen patients had one lung tumour, one patient had two tumours and three patients had three tumours. The median tumour volume was 2 cm^3^ (range: 0.3–102.1 cm^3^).

### 2.2 Abdominal compression and delineation

For compression the AC plate together with the stereotactic body frame (Elekta, Stockholm, Sweden) was used. The patients were positioned on a custom fitted vacuum pillow in the stereotactic body frame and the compression equipment consisted of an almost pentagon shaped compression plate of two different sizes and a compression screw of three different lengths. For each patient the most suitable plate and screw was used. The compression plate was placed just below the most caudal ribs and the screw was individually tightened to an acceptable level. The same level was used at both CT simulation and each treatment fraction. The patients were instructed to breathe as normally as possible with this forced shallow breathing. When constructing the custom fitted vacuum pillow, the stereotactic coordinates of both the position of the patient according to a tattoo on the sternum and the longitudinal position of the abdominal compression was set. At CT simulation and at each treatment fraction, the position of the patient and the abdominal compression was both adjusted to these stereotactic coordinates. Contrast was not used for any of the patients.

**Fig. 1:**
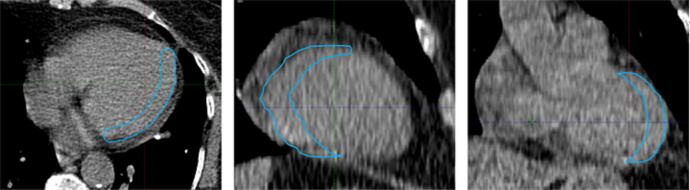
Example of delineation of the lateral left ventricle wall in axial plane (left), coronal plane (middle) and sagittal plane (right).

For each patient two 10-phase respiratory 4DCT scans in a Somatom Definition AS+ (Siemens Healthineers, Erlangen, Germany) was acquired. For respiratory signal generation the Sentinel (C-RAD Positioning AB, Uppsala, Sweden) was used during image acquisition. The voxel size in the images was 0.98 × 0.98 × 2 mm. The lung was automatically segmented in both 4DCTs in the Eclipse (v 15.6) treatment planning system (Varian Medical Systems, Palo Alto, CA, USA). The phase with the smallest lung volume was defined as maximum (max) expiration phase and the phase with the largest lung volume was defined as the max inspiration phase. In these two phases the following structures were delineated: the left coronary artery (LCA), the left anterior descending artery (LAD), the lateral wall of the left ventricle, the apex of the heart, the carina, and the right and left diaphragm. The LCA, LAD, carina and diaphragm were all delineated as small point structures, at first in the max expiration phase and thereafter in the same corresponding CT plane in the max inspiration phase. The heart apex was delineated in the two most inferior planes of the heart visible on the CT. The lateral wall of the left ventricle was delineated with the brush tool with a diameter of 1 cm, starting at the anterior top of the ventricle, down to the posterior part of the ventricle, along the lateral wall towards the lung (Fig. 1). The selection of these structures resulted from discussions between a radiation oncologist with clinical expertise in cardiac SBRT and a medical physicist. The delineations were carried out by the medical physicist, after detailed instructions and training with the radiation oncologist. After completing the delineation of all structures, the radiation oncologist reviewed the procedure and approved the structures.

### 2.3 Analysis and statistics

Using the statistics tool in Eclipse, the center of mass (COM) shift from max expiration phase to max inspiration phase was obtained in left–right (LR), anterior-posterior (AP), superior-inferior (SI) directions. The 3D vector length was calculated using $\sqrt{{\mathrm{LR}}^{2}+AP^{2}+SI^{2}}$. The size of this shift was compared between with and without AC. The Shapiro-Wilks test was used to test all data for normality. Since the data was not normally distributed for all structures, the median and range were evaluated, and the Wilcoxon signed rank test was used with a significance level of 0.05 for statistical comparisons.

## 3 Results

**Fig. 2:**
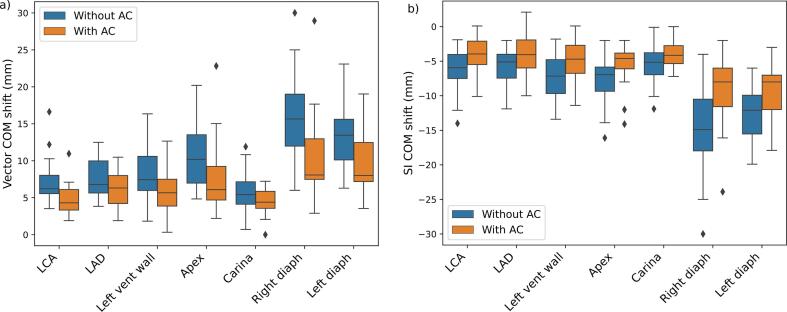
Boxplot of the vector center of mass (COM) shift (a) and the superior-inferior (SI) COM shift (b) for the left coronary artery (LCA), left anterior descending artery (LAD), left ventricle wall (left vent wall), the heart apex, the carina, and the right and left diaphragm (diaph). Blue boxes show the COM shift without abdominal compression (AC) and orange boxes with AC. The median is marked as the horizontal line within the boxes and outliers are presented as the black diamonds. (For interpretation of the references to colour in this figure legend, the reader is referred to the web version of this article.)

**Fig. 3:**
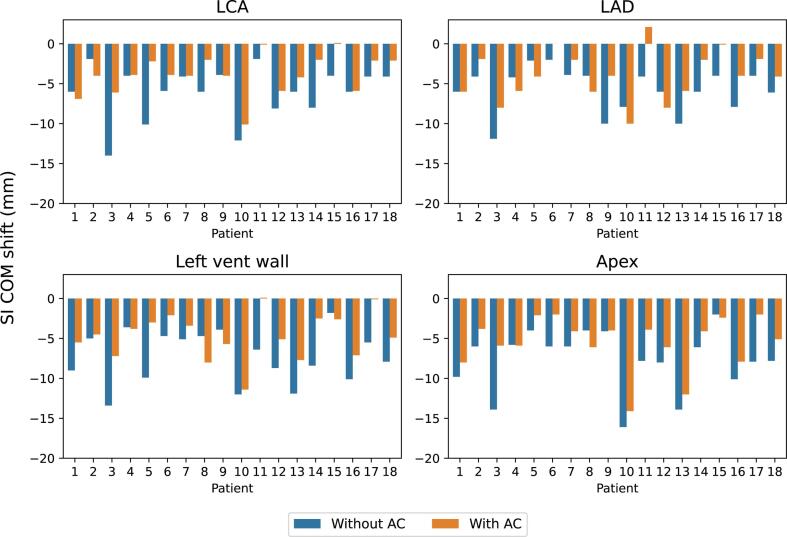
Center of mass (COM) shift from max expiration to max inspiration in 4DCT without abdominal compression (AC) (blue) and with AC (orange) in the superior-inferior direction for the left coronary artery (LCA), left anterior descending artery (LAD), left ventricle wall (left vent wall) and the heart apex. Negative values correspond to an inferior shift from expiration to inspiration. (For interpretation of the references to colour in this figure legend, the reader is referred to the web version of this article.)

The median 3D vector COM shift from max expiration to max inspiration phase was reduced by 1.9 mm for the LCA, 0.5 mm for LAD, 1.8 mm for the left ventricular wall, 4.1 mm for apex, 1.0 mm for carina, 7.6 mm for the right diaphragm and 5.5 mm for the left diaphragm (Fig. 2). Median COM shift LR, AP, SI, and 3D vector from max expiration to max inspiration for both with and without AC are presented in Table 1. The largest motion reduction was seen in SI direction, where for instance the median motion for the left ventricle wall was reduced from 7.2 to 4.7 mm (p < 0.01). For the left ventricle wall in SI direction, 4 out of the 18 patients had an increase in motion using AC, with 3.3 mm being the largest increase. The remaining 14 patients were all subject to motion reduction with AC, where 10 patients showed a large motion reduction (≥3mm), 2 patients had a moderate reduction (1–3 mm), and 2 patients had a small reduction (<1 mm). For all delineated structures, AC reduced motion in the diaphragm the most, implying that the AC was placed correctly on the patients. Statistically significant difference between with and without AC was found in SI only, for all structures but the carina (Table 1), indicating that the respiratory induced motion in the carina is not significantly affected by AC. Generally, the motion decreased when AC was used, however for some patients and structures, motion increased with AC (Fig. 3).

**Table 1:** The median center of mass (COM) shift from max expiration to max inspiration for the delineated structures, for both with and without abdominal compression (AC). Ranges are displayed in parenthesis. Statistically significant difference is marked with an asterisk. Negative values correspond to right, anterior and inferior shifts from expiration to inspiration for the left–right (LR), anterior-posterior (AP) and superior-inferior (SI) direction, respectively.

	LR (mm)			AP (mm)			SI (mm)			Vector (mm)		
	Without AC	With AC	*p*	Without AC	With AC	*p*	Without AC	With AC	*p*	Without AC	With AC	*p*
LCA	−0.2 (−8.9 - 4.9)	0.0 (−4.7 - 4.6)	0.25	0.1 (−2.3 - 4.5)	−0.1 (−3.8 - 1.9)	0.10	−6.0 (−14.0 - (−1.9))	−4.0 (−10.1 - 0.1)	<0.01*	6.2 (3.5 - 16.6)	4.3 (1.9 - 11.0)	<0.01*
LAD	0.8 (−5.0 - 9.6)	−0.1 (−5.6 - 6.9)	1.00	−0.5 (−9.3 - 4.6)	−0.6 (−4.1 - 6.4)	0.52	−5.1 (−11.9 - (−2.0))	−4.1 (−10.0 - 2.1)	0.01*	6.8 (3.8 - 12.5)	6.3 (1.9 - 10.9)	0.14
Left vent wall	−0.6 (−8.6 - 4.2)	−0.1 (−4.8 - 5.8)	0.37	−1.0 (−7.5 - 3.4)	−0.5 (−4.8 - 3.0)	0.18	−7.2 (−13.4 - (−1.8))	−4.7 (−11.4 - (0.1))	<0.01*	7.4 (1.8 - 16.3)	5.7 (0.3 - 12.7)	0.01*
Apex	−2.7 (−12.2 - 9.0)	−0.3 (−16.4 - 4.7)	0.89	−2.5 (−9.4 - 5.3)	−1.7 (−10.4 - 1.9)	0.58	−7.0 (−16.1 - (−2.0))	−4.6 (−14.1 - (−2.0))	<0.01*	10.2 (4.8 - 20.2)	6.1 (2.2 - 22.8)	0.01*
Carina	0.4 (−1.7 - 2.7)	−0.1 (−1.8 - 3.4)	0.44	0.0 (−3.9 - 2.3)	0.0 (−2.0 - 1.9)	0.48	−5.2 (−11.9 - (−0.1))	−4.2 (−7.2 - 0.0)	0.05	5.4 (0.7 - 11.9)	4.4 (0.0 - 7.2)	0.09
Right diaph	−0.2 (−5.7 - 2.0)	0.1 (−9.1 - 0.8)	0.49	−3.0 (−11.7 - 0.5)	−0.1 (−16.3 - 0.2)	0.13	−14.9 (−30.0 - (−4.0))	−8.0 (−23.9 - (−2.0))	<0.01*	15.7 (6.0 - 30.0)	8.1 (2.9 - 28.9)	<0.01*
Left diaph	−0.2 (−1.9 - 3.4)	0.0 (−3.9 - 1.1)	0.80	−0.6 (−11.2 - 0.6)	0.0 (−9.0 - 0.6)	0.06	−12.1 (−19.9 - (−6.0))	−8.0 (−17.9 - (−3.0))	0.01*	13.5 (6.3 - 23.1)	8.0 (3.6 - 19.0)	<0.01*

## 4 Discussion

In this study the effect of AC on respiratory induced motion in the heart was evaluated. Results showed a statistically significant reduction of motion in SI direction for all delineated heart structures. The results also indicate that AC could decrease the median respiratory motion of heart structures by approximately 1–3 mm. This relatively small effect could still be of clinical significance given the very high dose that is delivered in only one fraction in SBRT of VT, with vital organs in close proximity to the target. The heart motion reduction could allow for PTV margin reduction, however evaluation of random and systematic errors of the VT patient population should be carried out first. Our results suggest that approximately 60% of the patients will have large (≥3 mm) motion reduction in SI direction when AC is utilised. However, 4 of 18 patients had an increase in motion using AC, indicating the need for individual evaluation. By performing one 4DCT without AC and one 4DCT with AC for each patient, the individual benefit of AC could be assessed.

To our knowledge this is the first study to evaluate the effects of AC on respiratory induced motion in substructures of the heart. For instance, the motion of the left ventricle was examined, which is of particular interest for VT patients. Rasheed et al. [19] studied the effects of AC in lung cancer patients, including the motion of the whole heart. They could not show a significantly reduced motion for neither the heart nor the tumour. In our study significant motion reduction was found in all heart structures in SI direction. Rasheed et al. [19] did show that the same patient can exhibit motion reduction in one structure in the thorax, while another structure increases in motion, similar to our results (Fig. 3). This is another indication of why it would be beneficial to assess the effect of AC on an individual level for all VT patients who could tolerate the procedure.

In this study a straightforward comparison of motion with and without AC could be carried out since the data was paired. Since we did not have access to enough data for real VT patients, lung cancer patients were used as a surrogate cohort. The respiratory motion in the heart of a lung cancer patient might not be the same as in the heart of a patient suffering from VT. Nonetheless, we do still believe that these results give valuable information on how AC affects heart motion.

To be able to compare the motion of the heart structures with other surrounding structures, the diaphragm and carina were delineated as well. AC did not reduce motion in the heart as much as it did for the diaphragm, which is to be expected, since the diaphragm is the key muscle for respiration. The motion of the carina was not significantly affected by AC implying that the thorax superior to the heart benefit less from AC.

COM shifts are useful when studying small point structures, since the motion of COM closely corresponds to the motion of the structure. For larger structures however, such as the lateral left ventricle wall in this study, the COM motion may be less representative, since the ventricle deforms during both cardiac and respiratory cycle. Delineation on 4DCT phase images was challenging since it is more prone to motion artefacts than a regular CT. Moreover, the medial border of the left ventricle towards both the left atrium and the interventricular septum was also sometimes difficult to distinguish. Delineation uncertainties were therefore largest in the LR and AP direction in the left ventricle wall. To minimize delineation uncertainties, all structures were delineated by the same observer. Our results showed a statistically significant difference in motion between with and without AC in the SI direction, in which most respiratory induced motion occurs. Thus, despite delineation uncertainties these results do give an implication that AC could be beneficial for VT patients undergoing RT.

As this study examined only one 4DCT with AC and one without AC for each patient, the reproducibility of patient breathing pattern or AC placement was not assessed. The primary objective of the current study was to investigate the potential motion reduction in the heart achieved using AC. However, considering that reproducibility is important for maintaining the motion reduction, it would be necessary to include AC placement evaluation when studying systematic and random errors in a potential PTV margin reduction.

Although the majority of patients had a motion reduction of the heart using AC, there could be a risk of pushing the stomach towards the target volume with the AC. In the future, it would therefore be of interest to investigate whether the AC leads to dosimetric benefits as well.

In conclusion, abdominal compression significantly reduced respiratory induced motion in the heart in the SI direction. Our results showed that a majority of the patients had a large (≥3 mm) motion reduction in the left ventricle wall when AC was used. However, a few patients had an increased heart motion, indicating the need for individual evaluation of AC.

## CRediT authorship contribution statement

**Annika Mannerberg:** Conceptualization, Methodology, Formal analysis, Investigation, Data curation, Writing – original draft, Visualization. **Martin P. Nilsson:** Conceptualization, Methodology, Validation, Writing - review & editing. **Anneli Edvardsson:** Conceptualization, Writing – review & editing. **Kristin Karlsson:** Resources, Data curation, Writing – review & editing. **Sofie Ceberg:** Conceptualization, Writing – review & editing, Supervision, Project administration.

## Declaration of Competing Interest

The authors declare that they have no known competing financial interests or personal relationships that could have appeared to influence the work reported in this paper.
