# Supplementary material for: Abdominal compression as motion management for stereotactic radiotherapy of ventricular tachycardia
Source: Phys Imaging Radiat Oncol. 2023 Oct 7;28:100499. doi: 10.1016/j.phro.2023.100499 (PMC10585386; doi:10.1016/j.phro.2023.100499)
Supplement: Supplementary Data 1 [file mmc1.docx]

**Supplementary materials**

**Table S-1**. The sex, age, and CTV volume for each patient. The number of data points provided for the CTV volume represents the number of tumours. (F = female, M = male).

| Patient | Sex | Age (years) | CTV volume (cm^3^) |
| --- | --- | --- | --- |
| 1 | F | 73 | 7.0 |
| 2 | F | 66 | 1.8 |
| 3 | F | 68 | 2.1 |
| 4 | F | 81 | 0.6 |
| 5 | F | 60 | 3.7 |
| 6 | F | 67 | 0.3, 0.5, 0.8 |
| 7 | M | 79 | 6.6 |
| 8 | F | 81 | 1.4 |
| 9 | M | 76 | 0.6 |
| 10 | M | 66 | 102.1 |
| 11 | F | 78 | 2.6 |
| 12 | F | 76 | 10.8 |
| 13 | F | 62 | 0.3, 0.3, 18.0 |
| 14 | F | 82 | 5.0 |
| 15 | M | 80 | 0.6, 0.8, 1.5 |
| 16 | M | 51 | 12.4 |
| 17 | M | 75 | 2.0, 14.9 |
| 18 | M | 78 | 2.0 |
